# Mitochondrial metabolism and DNA methylation: a review of the interaction between two genomes

**DOI:** 10.1186/s13148-020-00976-5

**Published:** 2020-11-23

**Authors:** Amanda F. C. Lopes

**Affiliations:** 1 Department of Clinical Neurosciences, School of Clinical Medicine, University of Cambridge, Cambridge Biomedical Campus, Cambridge, CB2 0QQ UK; 2 Medical Research Council - Mitochondrial Biology Unit, University of Cambridge, Cambridge Biomedical Campus, Cambridge, CB2 0XY UK

**Keywords:** DNA methylation, Nucleus, Mitochondria, Metabolism, DNA, Haplogroups

## Abstract

Mitochondria are controlled by the coordination of two genomes: the mitochondrial and the nuclear DNA. As such, variations in nuclear gene expression as a consequence of mutations and epigenetic modifications can affect mitochondrial functionality. Conversely, the opposite could also be true. However, the relationship between mitochondrial dysfunction and epigenetics, such as nuclear DNA methylation, remains largely unexplored. Mitochondria function as central metabolic hubs controlling some of the main substrates involved in nuclear DNA methylation, via the one carbon metabolism, the tricarboxylic acid cycle and the methionine pathway. Here, we review key findings and highlight new areas of focus, with the ultimate goal of getting one step closer to understanding the genomic effects of mitochondrial dysfunction on nuclear epigenetic landscapes.

## Background

### Mitochondrial diseases and population prevalence

Mitochondria display the distinctive feature of being the only mammalian cellular organelle containing an independent genome, the mitochondrial DNA (mtDNA). This DNA can be found in different copy numbers depending on the cell and tissue type, rendering mtDNA as polyplasmic. Quantities can range from around 10^3^ to 10^4^ genomes per cell, yet still only representing 1% of the total cellular DNA [1]. Mammalian mtDNA is a circular double-stranded molecule of approximately 16.6 kb in size [1] that encodes only 37 genes, 13 of which are respiratory chain subunits and 24 being RNA components, such as tRNAs and rRNAs [1, 2]. It is estimated that mitochondria contain approximately 1500 different proteins, indicating that the vast majority of these are being encoded by the nuclear genome and imported into mitochondria [1]. Pathogenic mutations can occur in both nuclear DNA (nDNA) and mtDNA, with the mitochondrial genome presenting a mutational rate 100 fold higher than that of the nuclear genome, in turn leading to the heterogenous nature of inheritance of mitochondrial diseases [3]. Varying disease penetrance, as well as the occurrence and accumulation of spontaneous mutations in either genomes, contributes to an ever-expanding phenotypic spectrum [3]. Together, the variance in clinical expression and the multisystemic nature of these diseases has led to poor diagnosis and prognosis of mitochondrial diseases. These disorders cause significant morbidity and mortality, with a total of 1 in 4300 adults presenting with a mitochondrial ailment, and with these conditions being among the commonest inherited forms of neurological diseases [3]. Careful clinical and biochemical characterization of such pathological phenotypes is key for detection, discovery of effective treatments or cure of these debilitating diseases.

In the last few decades, advances in next-generation sequencing and non-invasive diagnostic methods have started to transform our understanding of mitochondrial diseases. Nevertheless, comprehending the complexities of the mechanisms behind these pathologies remains elusive. It is speculated that one layer of intricacy of varying mitochondrial disease penetrance could involve differential DNA methylation (DNAm). Genomic DNA methylation is a highly conserved mechanism that plays a pivotal role in development, tissue specification and diseases like cancer and neurodegeneration [4, 5], where methylation of certain cytosines can alter gene expression.

**Fig. 1:**
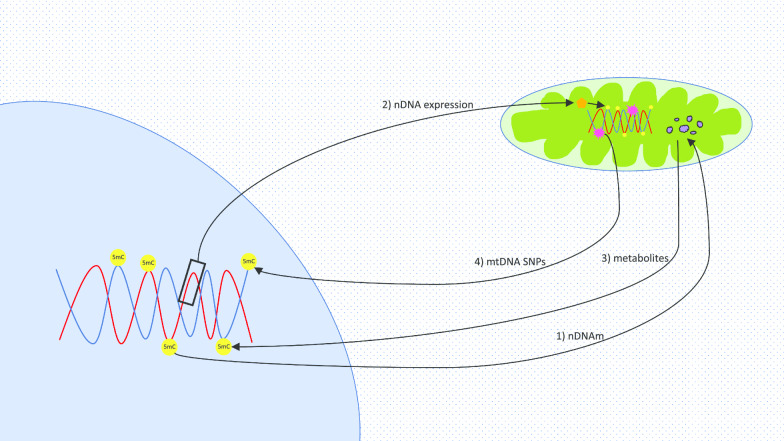
Interactions between DNA methylation and mitochondria. Arrows refer to different phenomena. (1) nDNAm: Nuclear DNA methylation impact on mitochondrial metabolism. (2) nDNA expression: Influence of nuclear gene expression on enzymes which may cause mtDNA methylation. (3) Metabolites: Effect of mitochondrial metabolites on nDNA methylation. (4) mtDNA SNPs: Burden of mtDNA mutations and haplogroups on nDNA methylation. Metabolites are presented in purple; DNA methylation sites are shown in yellow; DNA mutations are displayed in pink; orange refers to enzymes

Mitochondrial genomics and metabolism have been studied in the context of DNA methylation. Cells harbouring distinct mitochondrial haplogroups and mtDNA polymorphisms have been shown to present differences in nDNA methylation [6–8]. Moreover, as many metabolites are produced in the mitochondria but transit to the nucleus, alterations in their levels have been found to influence the efficiency of enzymes and affect the production of substrates required for methylation [9, 10]. The focus of this review is to discuss the relationship between mitochondria and the nucleus on an epigenetic level centred on DNA methylation (summarized in Fig. 1). Advances in the field shall be considered, remaining open questions will be proposed, and cause and effect versus association discussions have been instigated. Elucidating and debating these may be key to the development of future treatments, guide more specific clinical prognosis and form more comprehensive prevention strategies.

### What is DNA methylation and why is it important?

DNA methylation entails the conversion of cytosine to 5-methylcytosine (5mC), predominantly due to a DNA methyltransferase (DNMT) enzyme transferring a methyl group from S-adenosyl-methionine (SAM) to the 5th carbon of the ring of cytosine. This conversion is usually found within CpG dinucleotide sites (regions of the DNA in which a cytosine nucleotide is immediately followed by a guanine nucleotide in a linear sequence in the 5′ to 3′ direction [11]) despite occurrences also in non-CpG methylation sites [4]. Such epigenetic DNA modifications occur in intronic, exonic and intergenic regions [12], and are involved in the regulation of gene expression, either via interaction with promoters, enhancers, transcription factors and gene bodies, or via stimulating transcriptional elongation and gene splicing [12, 13].

With age, genome-wide levels of methylation are reported to generally decrease overall across multiple tissues (referred to as hypomethylation) [14, 15], but also specifically in certain tissues, e.g., in blood and brain [4], particularly in repetitive elements of intergenic regions [16]. Conversely, promoter-associated CpG islands present increased DNA methylation (known as hypermethylation) [17]. More explicitly, gene ontology (GO) analyses of genes with differential methylation due to aging have found hypermethylation to occur significantly in biological processes involved in cell-to-cell signalling, and development, while hypomethylation studies have shown enrichment in mechanisms correlated with mRNA and protein metabolism, immune responses, and mitosis [18].

In humans, alterations in DNA methylation levels in coding regions are associated with one-third of mutations that cause diseases in the germline [13, 19]. The mutational rate of methylated cytosines involving transitions to thymine has been shown to be more than 40-fold higher compared to random mutations. This suggests that DNA methylation can also contribute to the incidence of pathology via permanently changing the DNA sequence [15, 19]. Apart from its connection to developmental disorders, DNA methylation has been found to occur in many different human diseases originating due to somatic malignant changes, ranging from cancers to neurodegenerative and psychotic disorders (for a detailed review refer to [11]). Interestingly, DNA methylation also plays an important role in preserving genomic stability, as methylation of CpGs in transposons, retrotransposons and repetitive sequences prevent the expression of these elements [20].

The importance of nDNA methylation in disease penetrance is evident from studies of monozygotic twins, who share an identical genetic background, but distinct epigenetic alteration [21, 22]. Such investigations reveal clear phenotypic variations between the individuals, including that of predisposition to pathological conditions, such as neurodegeneration, cancer, and autoimmune diseases [20].

Global DNAm progressively diverges and changes during a person’s lifetime and ageing. Still, some changes in locus-specific DNAm sites have been found to be highly reproducible independently of gender and tissue being analysed, and hence can be used as a measure for age. Intrinsically, global DNAm levels have been recently described as a biomarker of chronological age. This biomarker estimate is known as the epigenetic clock or DNA methylation age [23], where measurements in sorted cells, tissues and organs [14, 24] are made independent of classic risk factors while still being able to predict all-cause mortality [23].

Ageing is characterized by an overall decrease in DNAm across most tissues. This global genomic hypomethylation has also been found in a number of different human cancers [25], as has locus-specific hypermethylation. A notable example is caused by increased DNAm in the promoter region of the gene *P16*—a common cause for development of human cancers due to epigenetic events [25]. Furthermore, differential DNAm correlates with neurodegenerative disorders with Alzheimer’s disease (AD) patients suffering from global hypomethylation [26, 27]. More specifically, studies have described inhibited DNMTs leading to cognitive impairment and disease progression [26].

Mitochondrial diseases are no exception to the list of human diseases speculated to have an epigenetic component, with examples involving pathologies caused by mtDNA mutations leading to LHON (Leber’s hereditary optic neuropathy) [28], and non-syndromic deafness due to the m.1555A > G mutation [29, 30]. While genetic in origin, some studies hypothesize on the involvement of an epigenetic component which can at times exacerbate these phenotypes. In many of such studies, the exact epigenetic link is still unclear, rendering these connections as of yet merely understood as environmental factors. This is the case for LHON in which environmental factors, often referred to as mutagenic elements, such as carbon monoxide [31], cigarette smoke, alcohol [28], and also the antituberculosis medication ethambutol [32] have been presented as components that can induce more severe symptoms for that specific disease and hence have the potential to be directly or indirectly epigenetic or genetic processes. To our knowledge, the mechanisms behind how environmental factors influence the onset or development of these disorders are still unknown. One conjecture could involve an epigenetic modification, such as DNAm of the mitochondrial genome affecting the expression and function of intramitochondrial enzymes, while another speculation might revolve around nuclear genes being influenced by epigenetic alterations leading to changes in mitochondrial functionality. Another mitochondrial disease debated to have an epigenetic component is non-syndromic deafness. Research in mice and human cancer cell lines have shown an association between rRNA hypermethylation, an activation of the proapoptotic transcription factor E2F1 and faulty mitochondrial biogenesis in respect to two mitochondrial regulatory proteins: the rRNA methyltransferase-related human mitochondrial transcription factors B1 (h-mtTFB1) and B2 (h-mtTFB2) [30, 33, 34]. Few mitochondrial diseases have been studied directly in relation to epigenetics, in particular DNA methylation, rendering this an important field open for exploration. It is likely that a limited number of studies around this topic are related to the complexity surrounding untangling mitochondrial diseases and metabolism, and understanding its cause or effect relationship to epigenetics. Constraints imposed by current technology, despite rapidly evolving over the last 20 years, imply that mtDNA sequencing as of yet cannot be carried out in parallel to methodologies to measure DNA methylation, at least not on a single cell level. Still, studies could begin with sequencing to measure DNAm and the mitochondrial genome on a multicellular tissue or cell type level, in either human mitochondrial disease patients or animal models. The latter presents advantages in terms of facilitating the maintenance of environmental aspects, and stability of nDNA backgrounds, and thus, encouraging longitudinal investigations. This could commence the detailed dissection of this relationship with the aim to define genes and proteins that may be involved in any causative links.

## Effects of nuclear DNA methylation on mitochondria

Nuclear DNA methylation can impact the expression level of nuclear-encoded genes and nuclear-encoded mitochondrial genes. While the former group of genes perform a variety of cytoplasmic, organelle-specific and even extracellular functions, the latter group of genes are translated into proteins and enzymes that are specifically required for mitochondrial transcription and replication, and the mitochondrial respiratory chain complex [35]. A seminal example of abnormally increased DNA methylation levels altering mitochondrial functionality is that of the nuclear-encoded mitochondrial gene DNA polymerase gamma (*POLG*)*.* In particular, methylation at the catalytic subunit A (*POLGA*), which is important for mtDNA replication and embryogenesis, has been negatively correlated with mtDNA copy numbers. This increased methylation was observed in differentiating embryonic stem cells and reflects the process of establishing the ‘mtDNA set point’ from which mitochondria expand in a cell-specific manner [36]. Likewise, DNA demethylation increases the expression of many factors involved in mtDNA replication like TOP1MT and POLG, consequently elevating the levels of mitochondrial copy numbers in glioblastoma cells [37]. Alterations in DNAm can additionally be found in genes associated with oxidative phosphorylation (OXPHOS) in disease or with age. Such is the case in human patients with autism spectrum disorder (ASD) presenting differences in methylation of the genes encoding for complex I, such as *NDUFA4*, *NDUFB2*, *NDUFB4* and *NDUFB6*, complex III, like *UQCRC2*, and complex IV, for instance *COX7B* [38]. Likewise, complex IV methylation alterations are also seen in non-diseased elderly human muscle tissue [39] and in laboratory high fat diet-induced insulin resistance rats [40], in the genes *COX7A1* and *Cox5a*, respectively. These studies illustrate the paramount impact nuclear DNAm has on mitochondria, in terms of mtDNA content and mitochondrial functionality.

## Is mitochondrial DNA methylated?

Since the 1970s, whether methylation occurs in the mtDNA has been a topic of debate [41–43]. Experimentation on frogs and HeLa cells indicated that mitochondria lack the machinery necessary for DNAm [41]. Nevertheless, measurements of 5-methylcytosine levels, the most abundant DNA methylation modification, have revealed that this epigenetic modification does indeed occur in the organelle but at lower levels than in the nucleus, ranging from one-fourth to one-fourteenth of that found in nDNA [42]. Still, different methodologies for measurements, including CpG- or non-CpG-specific sites, varying mtDNA regions assayed, including the L- (light) and H- (heavy) strand composition of mtDNA, and the differing cell types investigated have predicted mtDNA methylation approximations to range from 1 to 20% [44]. Several studies mainly employing bisulfite sequencing in human [45] and in mouse [46] have questioned the presence of mtDNA methylation, implying, for example, that secondary structures of mtDNA and its circular structure may be causing overestimations of methylation signals [44]. Consequently, while some claim that mtDNA methylation levels are too low to be considered significant and current techniques are too biased to be reliable, others claim that mtDNA is methylated differently to nDNA, in this case in a strand-specific manner [15], only in non-CpG sites, yet at quantifiable levels [15, 47]. Notwithstanding, this topic remains highly debated.

Despite the fact that functional roles of mtDNA methylation remain unknown [35], other nuances have been detected. Absolute quantities of CpG dinucleotides in mtDNA relative to other species have been reported to be smaller than expected if there were no factors influencing these CpG sites [48]. Additionally, specific patterns of CG dinucleotide rich areas can be seen in the mtDNA, with as many as 50% of these being found in polymorphic variants. This uneven distribution raises questions regarding the possibility that mtDNA methylation might have taken place.

Still, DNMTs and ten-eleven translocation (TETs), which are enzymes instrumental in DNA methylation and demethylation, respectively, usually found in the nucleus, have been spotted in the mitochondria, and present an apparent influence on mtDNA methylation. DNMT1 has been observed to translocate into the mitochondria and interact with the mtDNA in the matrix of some tissues such as mouse embryonic fibroblasts, human colon carcinomas [35], and human brain cells [49]. In fact, *DNMT1* mutations can lead to development of two neurodegenerative diseases known as autosomal dominant cerebellar ataxia-deafness and narcolepsy (ADCA-DN) and hereditary sensory neuropathy with dementia and hearing loss (HSN1E). Importantly, both syndromes display clinical features typical of known mitochondrial diseases, like optic atrophy, peripheral neuropathy, deafness, and mitochondrial dysfunction [50]. Interestingly, some nuclear encoded proteins involved in mitochondrial function, such as PGC1a and NRF1 (acting in a complex or alone), or P53, can up-regulate or down-regulate, respectively, the levels of intra-mitochondrial DNMT1 (mtDNMT1) [35]. These genes can also be found to be methylated, with *PGC1a *modifications affecting mitochondrial density in type 2 diabetic patients [51, 52], and *NRF1* methylation causing *TFAM* (mitochondrial transcription factor A) silencing and a reduction in mitochondrial biogenesis [53]. In fact, methylation of *PGC1a* can additionally be modulated by nuclear DNMT3 [52] or DNMT1, the latter of which can be inhibited by AMPK (AMP-activated protein kinase) phosphorylation [54]. These studies raise the question of whether differential methylation of nuclear genes controlling the expression and activity of *DNMT1* may also indirectly lead to changes in mtDNA methylation.

DNMT1 is not the only DNMT to be found in the mitochondria. While studying ALS (amyotrophic lateral sclerosis) in mouse, another neurological disease affecting voluntary muscle movements, researchers have found the presence of the enzyme DNMT3A in the CNS (central nervous system), striated muscle and testes of these animals, and reinforced these findings by detecting the same enzyme in mitochondria of human cerebral cortex [49, 55]. Similar results were also seen in another study which discusses the expression of the same gene in mitochondria of mouse and human CNS [49]. Levels of *Dnmt3a* were found to be significantly lower in skeletal muscle and spinal cord of these mouse models presenting early disease signs, and these appear concomitantly to abnormal patterns of DNA 5mC [55]. Nonetheless, DNMT3A is not the only DNMT3 found to act in mitochondria. DNMT3B can modify the frequency and quantity of mtDNA methylation in healthy breast cells, albeit in a strand specific manner, affecting the L-strand more significantly. The importance of DNMT enzymes was further confirmed in knockdown studies with *DNMT3B* and *DNMT3A*, leading to reductions in global mtDNA methylation [47] and regional mtDNA methylation [15], respectively. Additionally, when comparing the effects of knocking down *Dnmt1*, *Dnmt3a* and *Dnmt3b* in mouse embryonic stem cells, all of these presented an analogous mtDNA methylation pattern while remaining lower than wild type samples [56].

When discussing the presence of DNMT enzymes in the mitochondria, it is worth noting the isoform of the enzyme. While earlier reports [35] inferred that DNMT1-isoform1 localized to the mitochondria, a more recent study contradicts such claim and instead puts forward DNMT1-isoform3 as being able to translocate to the mitochondria and methylate the mtDNA [57]. In fact, different isoforms also play a role in the presence of DNMT3A in mitochondria, as shorter isoforms like 78 kDa *Dnmt3a* can be found expressed primarily in skeletal muscle, while longer isoforms of 100 kDa are found expressed mostly in the nervous tissue [55]. These results suggest that the presence of these enzymes and the differing levels of mtDNA methylation are tissue specific. It might be postulated that the presence of these enzymes required for this epigenetic change, particularly in specific tissues, might be functionally linked to the expression of specific mitochondrial genes, raising the question of whether mtDNA methylation could regulate certain genes in accordance with cellular and metabolic demand.

Other enzymes involved in modulation of 5mC in mtDNA have been detected in mitochondria too, namely TET enzymes. Despite unknown mechanisms of translocation to the mitochondria, TET1 and TET2 enzymes have been found to be present in mouse neuronal mitochondria, for example in the cerebellum and Purkinje cells of aged animals [58]. Moreover, the expression of *Tet2* and *Tet3*, once increased, is associated with increased levels of 5hmC (5-hydroxymethylcytosine) found both in the nuclear and mitochondrial DNA [58, 59]. While providing indications that TET enzymes could have a role in mtDNA methylation, the exact functioning of such enzymes is yet to be elucidated.

Recent studies have started to focus on addressing concerns of measuring mtDNA methylation raised in a number of investigations [38–41]. New methodologies have been presented which are specifically adapted to mitochondrial genomes to improve bisulfite conversion of mtDNA [47], and adapted to the analysis and methylation calling for this particular genome [15], yet do still acknowledge the limitations of some of these adaptations. One such example involves the fragmentation of the mtDNA via sonication in order to overcome secondary structures which could skew bisulfite conversion. Despite this adjustment, the technique produces a range of sizes of DNA fragments, many of which can be found in the small range of 100-200 bp, hence becoming prone to degradation, causing important loss of information [47]. Another study suggests that biases found in mtDNA methylation calling are not caused intrinsically by bisulfite treatment, but rather due to an inherent bias in the L- and H-strands of the mtDNA, likely due to the former strand carrying 12 of the 13 protein-coding genes of the mitochondria, backing up these claims by carrying out analyses also using a non-bisulfite method [15].

Still, despite the much-heated contrast of opinions on the topic, this discussion should not derail from the potential debates that could stem off the possibility that mtDNA methylation does indeed happen. Could potential DNMT and TET-modulated mtDNA methylation influence the expression levels of mitochondrial-encoded genes and their respective functionalities? If so, could this phenomenon loop back into affecting the nucleus and its DNA? Further interrogation of these questions could shed light on disease penetrance and phenotypic variability of mitochondrial diseases.

## Effects of mitochondria on nuclear DNA methylation

### Effects of mitochondrial DNA

Signals from the mitochondria to the nucleus are referred to as ‘the retrograde response’ and can control the expression of nuclear genes in order to regulate mitochondrial functionality and metabolism [2]. Recently, research suggested that mtDNA variants play a part in the link between mitochondria and nuclear DNAm. In studies using peripheral adult human blood, articular cartilage, and human retinal cell cybrids, which possess identical nuclei but different mtDNA, diverse haplogroups have been shown to present differing degrees of nDNA methylation [6, 7, 60]. This is the case for haplogroup J which has consistently been associated with higher levels of DNA methylation when compared to other haplogroups such as H in cybrids and cartilage cells [7, 60], and also U, X and T in blood [6]. Likewise, mtDNA haplotypes of mouse embryonic stem cells also present distinct DNAm patterns [61]. Haplotypes are described as a group of DNA variations that are inherited together and have accumulated with time due to maternal inheritance and the lack of recombination, while haplogroups refer to mtDNA polymorphism variations that are found in a group of similar haplotypes. Additionally, this has been observed in brains of mouse models containing identical nDNA yet varying mtDNA polymorphisms [62]. Removal of mtDNA in Rh_0_ cells further confirms these results, where abnormal methylation patterns in nDNA genes were partially restored to normal with re-insertion of the mitochondrial genome [63], thus suggesting that disruptions or mutations in mtDNA can result in epigenetic changes in nDNA. Future insights into the interplay between mtDNA and nDNA methylation could involve the study of mitochondrial heteroplasmy. In fact, recent findings suggest that epigenetic histone methylation is regulated by mtDNA heteroplasmy [64, 65]. Defining the mechanisms involved in such phenomena could be important to determine mitochondrial disease penetrance.

Since mtDNA-depleted (Rh_0_) cells exhibit minimal amounts of ATP (adenosine triphosphate) and a decline in nDNA methylation [6], and similarly, cybrid cells of the J haplogroup present lower levels of ATP compared to non-J cybrids [6, 8]; it has been claimed that ATP levels regulated by mtDNA are correlated with a decrease in global DNAm [6]. Studies indicate that the reduced ATP levels observed in the J haplogroup could be associated with problems in the mitochondrial respiratory complex. Variants clustered in the J haplogroup often fall within complex I (ND1) and complex III (cytochrome b), with some groups reporting single nucleotide polymorphisms (SNP) causing loss of structural integrity of ND1, a drop in oxygen consumption, or partial uncoupling of OXPHOS [6]. As such, J haplogroup has been found to have functional consequences on several complex traits like LHON, multiple sclerosis, neurodegeneration and longevity [66]. Haplogroup J has also been associated with significantly lower levels of MBD2 in comparison with haplogroup H [7]. This gene is part of a family of proteins that bind to methylated DNA and block transcription, ultimately, being associated with a number of conditions such as cancers [7]. Differential expression of these genes could also lead to variation in nuclear DNAm and transcription and subsequently affect inflammation pathways, which are activated in certain diseases, for example leading to the higher incidence of age-related macular degeneration seen in J haplogroup patients [7]. Similarly, other mitochondrial haplogroups have also been associated with increased risk of a number of different traits. Yet still, an in-depth study of the relationship between the J haplogroup and DNAm is lacking. Moreover, detailed studies on the impact of other haplogroups on DNA methylation are also limited.

Finally, the impact of mitochondrial respiratory complex dysfunctions on DNAm is starting to appear. Notable examples are seen in studies where rotenone-induced complex I dysfunction resulted in global changes in DNAm levels in rats, human cybrid cells, and even when induced in mother mice and measured in the offspring [64, 65, 67, 68]. Together, these studies enforce the notion that differences in mtDNA are signalled to the nucleus, influencing its DNA, and are consequently associated with nuclear DNAm [6, 69]. Whether these associations are correlative or causational remains to be elucidated. Further investigations on global and regional changes in DNAm using other drugs known to affect the mitochondrial respiratory complex, as well as studies involving animal models with mtDNA mutations for interrogating mitochondrial diseases, are warranted to initiate forging the way towards understanding the cause and effect link.

### Effects of mitochondrial metabolites

**Fig. 2:**
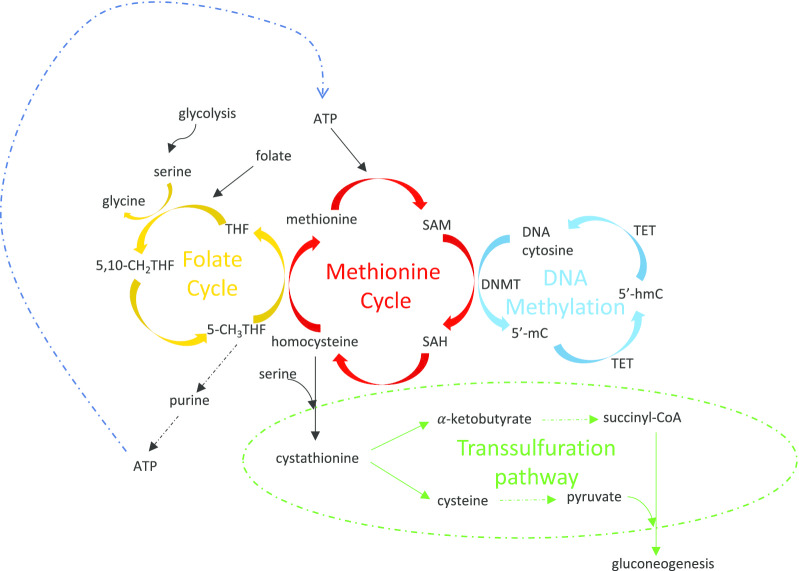
Metabolic processes tightly intertwined with DNA methylation. The yellow pathway refers to the folate cycle which mainly takes place in the mitochondria, and generates methionine. The red cycle is the methionine cycle which is important for the production of SAM in the cytoplasm. The blue network represents DNA methylation, and highlights the modification that happens on the genome, for example, in the nucleus. The transsulfuration pathway shown in green involves the irreversible transformation of homocysteine, leading to gluconeogenesis which takes place in the mitochondria, cytosol and the endoplasmic reticulum

Mitochondrial haplotypes and haplogroups affect a variety of signalling pathways. Some studies imply an effect of these variants on nuclear DNAm and suggest that this could be due to mitochondria influencing pathways such as OXPHOS, the methionine cycle [6, 8, 61], inflammation and angiogenesis [7]. In fact, recent findings suggest that mitochondrial dysfunction and mtDNA variations can cause alterations in metabolite levels, consequently interfering with nuclear DNAm. A tight interlinked network of metabolic pathways affecting DNA methylation involves the mitochondria (Fig. 2). These include, but are not restricted to: serine biosynthesis, the folate cycle, the methionine cycle, the transsulfuration pathway, and the Krebs cycle.

The folate cycle, also known as the one-carbon (1C) cycle, which mainly takes place in the mitochondria, entails the transfer of one carbon from either serine or glycine for the formation of DNA and RNA building blocks, or the generation of methionine. Subsequently, this pathway is linked to the methionine cycle, found in the cytoplasm, which is involved in the production of SAM, a methyl donor that is used for numerous reactions, including DNAm [70]. SAM is produced by methionine adenosyltransferase reacting with methionine and ATP. Once having lost its methyl group, SAM becomes SAH (S-adenosyl-homocysteine), which is converted into homocysteine, and can later be turned back to methionine, restarting the cycle, or feed into the transsulfuration pathway where it becomes irreversibly transformed [71]. The latter leads to the production of cysteine and α-ketobutyrate, both necessary for gluconeogenesis, a metabolic process that makes glucose from non-carbohydrate carbon sources. Interestingly, the levels of SAM have been found to be dependent on the mitochondrial haplogroup. One such example is seen in human cybrid cell lines of haplogroup J cells which have a higher expression level of methionine adenosyltransferase 1A (*MAT1A*), hence elevated levels of global methylation, when compared to the same cell types with H haplogroup [6].

**Fig. 3:**
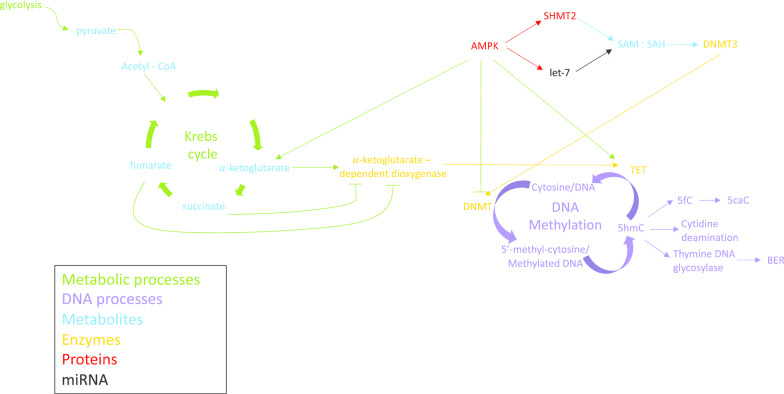
Interaction of Krebs cycle intermediates and AMPK with DNA methylation. Diagram shows how cytoplasmic glycolysis connects to the Krebs cycle, which occurs in the mitochondria. The latter affects the levels of TETs that are involved in DNA methylation, and which are regulated also by AMPK. AMPK modulates DNA methylation via DNMT directly and indirectly, and by interacting with the Krebs cycle. Different colours refer to the terms metabolic processes (green), DNA processes (purple), metabolites (blue), enzymes (yellow), proteins (red) and miRNA (black)

Antagonistic to this mechanism is glycolysis, a process that uses cytoplasmic glucose for the production of energy together with the Krebs cycle, which takes place in the mitochondria. The latter pathway uses acetyl-CoA, which is produced from the end product of glycolysis known as pyruvate, and undergoes a series of modifications producing intermediates like α-ketoglutarate, succinate and fumarate (Fig. 3). These metabolites all influence the activity of TET enzymes, which in fact belong to the family of α-ketoglutarate-dependent deoxygenases [9], and hence, require α-ketoglutarate to function. Succinate and fumarate, on the other hand, as seen in multiple human cancerous cell lines, are inhibitors of TET enzymes, and in the case of fumarate, it is capable of modulating TET also via reducing mRNA expression of *TET1* and *TET2*, though increasing mRNA expression of *TET3* enzymes [10]. Additionally, the activity of TET enzymes also changes in response to AMPK-mediated phosphorylation (Fig. 3). This is in turn dependent on glucose levels in mice and humans, with hyperglycaemic conditions impeding the phosphorylation, thus altering the quantities of 5hmC, the first oxidative product of the demethylation of 5mC [72]. Additionally, AMPK can also enhance the expression of TET enzymes directly or via increasing isocitrate dehydrogenase 2 (IDH2), a mitochondrial enzyme found in the Krebs cycle involved in the production of α-ketoglutarate, and hence activate TETs to ultimately decrease DNAm. Interestingly, AMPK plays a dichotomous role when it comes to DNMT enzymes. While it presents an inhibitory role towards DNMT1, it can also have a stimulatory effect on DNMT3s. The latter takes place as AMPK either transactivates let-7 microRNA which leads to an increase in the SAM/SAH ratio, or it activates serine hydroxymethyltransferase 2 (SHMT2) which converts serine to glycine in the mitochondria, facilitating production of SAM [54] (Fig. 3). In both scenarios, increased SAM activates DNMT3s, thus altering normal methylation patterns. This has been shown to be the case, in humans after exercise, for the methylation of promoters of genes like *COX4I* (cytochrome C oxidase subunit 4I1) [54]. This gene plays an important role in the transfer of electrons from complex III to complex IV in the mitochondrial respiratory chain; and exemplifies how mitochondrial metabolism can impact nuclear DNAm, and subsequently mitochondrial functionality.

Given the interlinked relationship of these pathways, alterations to any one or many of these processes can affect DNAm levels. An unbalance in the 1C metabolism has the potential to impact cell cycle, transcription, replication and signalling via the regulation of nucleotide pools, redox and methylation status, although the precise mechanism coming from the mitochondria remains elusive. Similarly, little is known about how mitochondrial dysfunctions bring on modifications in the mitochondrial folate pool and serine biosynthesis, despite the involvement of mammalian target of rapamycin (mTOR) and the activating transcription factor 4 (ATF4) [73]. In human cells with reduced mtDNA or during experimental respiratory chain blockade [73], ATF4 has been shown to be responsible for increasing the expression of genes involved in the production of serine, in an attempt to maintain the 1C pathway functioning. ATF4, once activated by mTOR, also has the ability to regulate the mitochondrial folate pool in cells with mitochondrial myopathy [74]. In these same mammalian cells, mTOR can additionally coordinate the induction of 1C-dependent pathways, including the folate cycle, serine biosynthesis, transsulfuration, and dNTP synthesis [74]. Alongside this, loss of mtDNA causes perturbations to the Krebs cycle, igniting a response in the methionine and polyamine metabolism separately, perhaps due to their contributions to succinate [75], but also impacting the methionine cycle through the polyamine metabolism, independently of serine-driven 1C metabolism or transsulfuration [75]. Moreover, depletion of mtDNA can lead to the activation of serine biosynthesis, folate pool remodelling, and to a shift in the 1C metabolism [63] towards transsulfuration, thus leading to the generation of cysteine that will be used for gluconeogenesis [75]. While precise mechanistic links between these pathways are still missing, current studies pinpoint to the complexity of the level of intertwining between these cycles. Likewise, the Deletor mouse strain lacking the mtDNA helicase Twinkle, and hence deficient in replication, also presents alterations in these pathways. In this scenario, the metabolic changes were found to be due to increases in the levels of choline and betaine, which are involved in the conversion of homocysteine to methionine, and ornithine that later becomes methylthioadenosine (MTA) also involved in the regulation of methionine [76].

Methionine is crucial for DNAm due to its requirement for the production of SAM, which consequently is needed by DNMT for the methylation of cysteine residues on DNA. As an essential amino acid [77] with high significance in DNAm, it has previously been speculated that increasing its dietary intake could increase DNAm [78]. In fact, methionine supplementation in rats influences tissue-specific nDNA methylation levels, indicative of a non-uniform response to this 1C metabolic substrate [79]. While confirming an involvement of methionine in nDNA methylation levels, further research is required to better understand the extent of its involvement and whether it could indeed be used to reverse DNAm alterations in the clinic [80].

In the absence of methionine in human colorectal cancer cells, the serine biosynthesis pathway provides one-carbon units to the methionine cycle, allowing homocysteine to be converted to methionine. In parallel, regardless of the methionine status, the serine cycle is involved in DNAm as it is important and rate-limiting in de novo ATP synthesis which is required for the generation of SAM from methionine [70]. The way in which the serine biosynthesis pathway divides its consumption of one-carbon units between nucleotide synthesis, more specifically purine, and homocysteine remethylation, can be controlled by serine hydroxymethyltransferase (SHMT1). The demand for serine is met via a combination of exogenous serine uptake and generation from intracellular glucose. Like serine, other amino acids and metabolites maintain their levels stable by diet and cellular metabolism, as is the case for methionine. Yet, as previously described, simply altering just one factor is an underestimate of its impacts, since methionine has also been classified as the most toxic amino acid and can lead to the formation of methanethiol-cysteine disulfides that cause methionine toxicity if supplied in excess to small animals like chickens, rats and quails [81].

Collectively, the current state of research on the interaction between mitochondrial metabolism and nuclear DNAm highlights a number of gaps in our knowledge. This is likely due to the complexity of the various pathways, how tightly interlinked they function, and the fact that some genes have various roles regarding DNAm, some of which are dichotomous. Moreover, it is apparent that DNAm and metabolic differences are organism-, tissue- and cell-type specific, perhaps due to the inherently different mitochondrial needs of each subtype, which might stem off which donors for the 1C are used, and how 1C substrates are metabolized in every case [73, 82]. Different techniques are currently employed in an attempt to unravel such complexities, including but not limited to liquid chromatography and mass spectrometry, multi-omics like proteomics and metabolomics, and most recently, MITO-Tag which is available in vitro as well as in vivo for mice, allowing for the rapid isolation of cell-type specific mitochondria from tissues [83]. Despite these novel approaches, untangling this synergy as of now, can only be considered as the beginning of a long, exciting process.

## Conclusions and future perspectives

Limited treatments for mitochondrial diseases due to its wide spectrum of clinical phenotypes and variable penetrance have led to the development of mitochondrial replacement therapy (MRT). This technique has enabled mothers that are carriers of mtDNA mutations leading to debilitating mitochondrial syndromes to have children without transmitting their maternal disease-causing mtDNA. Despite the immeasurable possibilities this procedure has provided, it also raises concerns about the reciprocal interaction between the nucleus and mitochondria. While the efficient replacement of mutant mtDNA in oocytes have resulted in embryos with > 99% of donor mtDNA, some embryonic stem cells derived after the therapy have demonstrated a gradual loss of the donor mtDNA, presenting a reversal to the original mitochondrial haplotype observed in the oocyte pre-treatment [84]. Incompatibility of nuclear and mitochondrial genomes is not a new topic, leading to impacts on the physiological fitness of the offspring shown for example in studies carried out in yeast, *Drosophila* and mice [85], although not yet seen in humans and non-human primates [86]. Epigenetic factors like DNA methylation have not yet been addressed in this context. While MRT remains an uncommon treatment, further investigations into the interplay between the newly replaced mitochondria and the oocyte’s nucleus, including DNA methylation, may aid in the prevention of unanticipated caveats in future newborns.

DNAm is implicated in a wide range of diseases including cancer, diabetes, neurodegenerative disorders, and mitochondrial pathologies. A multitude of techniques have been employed to study this link, ranging from methylation-specific PCR and pyrosequencing, to methylation arrays and whole genome bisulfite sequencing. Still, results remain correlative instead of causative. While correlative studies are important, in order to interrogate causational links, recent investigations have started to use statistical inference to assess causality of their experiments. Mendelian randomization, a method that uses measurements of genes of known functionality to examine the causal effect of another putative variable for a given disease, has become one of the most commonly used analysis to enable unbiased estimates. Proving the usefulness of this approach, causality of DNAm of specific CpG sites has been linked to increased risks of disorders like cardiovascular disease, type 2 diabetes, and Parkinson’s disease [87].

Development of single-cell DNAm techniques alongside the possibility to use cells from formalin-fixed and paraffin-embedded tissue directly from patients has also offered potential for growth of this field [88]. The use of individual cells has created the opportunity not only to assess heterogeneous systems and rare cell types, but has generated the ability to use very low input materials, hence bringing more clinical applications within reach. This fast-developing technology has allowed for the simultaneous analysis of multiple aspects of a mere cell including, but not limited to: gene expression, DNAm, chromatin accessibility, copy number variation, and genotype [89]. This young field has opened the possibility to integrate different layers of information with the promise to create a clearer picture of the role of DNAm, for example, in reference to mitochondrial diseases and aging.

Aging is also highly correlated with mitochondrial function, as an accumulation of mtDNA mutations can lead to a reduction in functionality of the mitochondrial respiratory chain. In fact, studies carried out on the mtDNA mutator mouse which harbour a number of different somatic mutations, show that these animals present various aging phenotypes such as osteoporosis, weight reduction, hair loss, greying of hair, and loss of fertility [90]. Importantly, the majority of human age-related diseases have a mitochondrial component to them, in particular neurodegenerative diseases. Recent developments in measuring age through DNAm have created the opportunity to investigate these three seemingly independent elements: mitochondrial parameters, DNAm profile, and age. Chronological and biological age (measured via different DNAm age clocks [91]) from tissues and most importantly blood samples of mitochondrial diseased patients can be studied in terms of DNA methylation, making personalized medicine, and preventive care, now, a more realistic proposition. Together, these three elements may create a holistic picture that could lead to the better understanding of mitochondrial diseases, improved diagnostic abilities and more tailored therapeutic strategies.

## Data Availability

Not applicable.

## Abbreviations

1C metabolism: 1 Carbon metabolism
5hmC: 5-Hydroxymethylcytosine
5mC: 5-Methylcytosine
AD: Alzheimer’s disease
ADCA-DN: Autosomal dominant cerebellar ataxia-deafness and narcolepsy
ALS: Amyotrophic lateral sclerosis
ASD: Autism syndrome disorder
ATP: Adenosine triphosphate
CNS: Central nervous system
DNAm: DNA methylation
GO: Gene ontology
HSN1E: Hereditary sensory neuropathy with dementia and hearing loss
LHON: Leber’s hereditary optic neuropathy
MRT: Mitochondrial replacement therapy
mtDNA: Mitochondrial DNA
MZ: Monozygotic twins
nDNA: Nuclear DNA
OXPHOS: Oxidative phosphorylation
Rh_0_: Cells lacking mitochondria
SAH: S-adenosyl-homocysteine
SAM: S-adenosyl-methionine
SNP: Single nucleotide polymorphism
